# Leveraging NHANES database for sleep and health-related research: methods and insights

**DOI:** 10.3389/fpsyt.2024.1340843

**Published:** 2024-04-30

**Authors:** Yanwei You, Yuquan Chen, Mengxian Wei

**Affiliations:** ^1^ Division of Sports Science & Physical Education, Tsinghua University, Beijing, China; ^2^ School of Social Sciences, Tsinghua University, Beijing, China; ^3^ School of Public Health and Preventive Medicine, Faculty of Medicine, Nursing & Health Sciences, Monash University, Melbourne, VIC, Australia

**Keywords:** NHANES, cross-sectional study, sleep, methods, insights

## 1 Introduction

In the modern society, where the pace of life is unrelenting and stress levels are escalating, sleep-related issues have garnered significant attention (1–4). Sleep, intricately linked to overall health, plays a pivotal role in psychological, physiological, and cognitive functions. Leveraging the National Health and Nutrition Examination Survey (NHANES) database for sleep-related research has emerged as a prominent area of academic interest. Current studies have explored the association between sleep and different health outcomes, such as mortality (5), aging process (6), inflammation (7), metabolic health (8), cardiovascular function (9), cognitive performance (10), and mental health (11).

However, a great number of world sleep researchers, especially doctors in the sleep medicine field, are not aware of the value of NHANES in exploring sleep health. One of the primary hurdles researchers face when delving into sleep health using the NHANES database is the lack of comprehensive knowledge about the sleep-related variables it contains. The database encompasses an extensive array of health-related data, making it challenging for researchers to pinpoint which specific variables pertain to sleep. Another significant challenge lies in the uncertainty surrounding the distribution of sleep-related variables across different survey years within the NHANES database. Given that not all sleep variables are present in a single year cycle, choosing the appropriate year to search for specific variables of interest holds significant importance. Finally, understanding the relationship between sleep-related variables and various health indicators presents a challenge. Researchers may encounter difficulties in designing studies that effectively tease out the influence of sleep on specific health indicators, given the multitude of confounding variables that may be at play.

This study aims to summarize key research methods and insights for utilizing the NHANES database in exploring sleep and its associated health indicators. Firstly, it seeks to uncover sleep-related variables pertaining to sleep duration, quality, and disturbances. Secondly, it aims to compile information on the distribution of these variables across different survey years.

## 2 Methods

NHANES, conducted by the National Center for Health Statistics (NCHS), is a nationwide and cross-sectional initiative aimed at assessing the health and nutritional status of the American populace (12). NHANES collects data from the non-institutionalized civilian population and releases this information in two-year cycles. This extensive database encompasses diverse participants from various racial, age, and socio-economic backgrounds, ensuring its broad representativeness and applicability.

In this section, we summarize tools and methods for sleep assessment in different year cycles of NHANES 2005-2018. The sleep questions were conducted at participants’ homes during the household interview using the Computer-Assisted Personal Interviewing (CAPI) system (2). Within this database, data related to sleep are primarily collected through the following methods, which is shown in **Table 1**.

**Table 1:** Distributions of sleep-related variables in NHANES.

	Year cycle 2005-2008	Year cycle 2009-2010	Year cycle 2011-2014	Year cycle 2011-2016	Year cycle 2017-2018
**Sleep Duration**	How much sleep do you get (hours)?	How much sleep do you get (hours)?	How much sleep do you get (hours)?	Sleep hours	
	How often did you not get enough sleep?				
					Sleep hours - weekdays or workdays
					Sleep hours - weekends
**Sleep Disturbances**	Ever told doctor had trouble sleeping?	Ever told doctor had trouble sleeping?	Ever told doctor had trouble sleeping?	Ever told doctor had trouble sleeping?	Ever told doctor had trouble sleeping?
	Ever told by doctor have sleep disorder?	Ever told by doctor have sleep disorder?	Ever told by doctor have sleep disorder?		
	How often wake up during night?				
	How often do you snore?			How often do you snore?	How often do you snore?
	How often do you snort / stop breathing?			How often do you snort / stop breathing?	How often do you snort / stop breathing?
	Sleep disorder: sleep apnea				
	Sleep disorder: insomnia				
	Sleep disorder: restless legs				
	Sleep disorder: other				
**Influence of Sleep Disturbances**	How often feel unrested during the day?				
	How often feel overly sleepy during day?			How often feel overly sleepy during day?	How often feel overly sleepy during day?
	How often have leg jerks while sleeping?				
	How often have legs cramp while sleeping				
	Difficulty concentrating when tired?				
	Difficulty remembering when tired?				
	Difficulty eating when tired?				
	Difficulty with a hobby when tired?				
	Difficulty getting things done?				
	Difficulty with finance when tired?				
	Difficulty at work because tired?				
	Difficulty on phone when tired?				
				Usual sleep time on weekdays or workdays	Usual sleep time on weekdays or workdays
					Usual sleep time on weekends
				Usual wake time on weekdays or workdays	Usual wake time on weekdays or workdays
					Usual wake time on weekends
**Accelerometers (PAM)**			Day of the week measurement started		
			The last day when the measurements taken		
			First data timestamp in GT3X		
			Timestamp at end of last day		
			Total seconds/minutes with data in the minute file or in the hour/day files		
			Total number of 80 Hz acceleration measurements logged during idle sleep mode		
			Total number of valid minutes in the hour/day		
			Predicted wake/sleep/non-wear status during the minute		
			Transition flag for wake/sleep/non-wear		
			Valid wake/sleep/non-wear/unknown minutes in the hour		
			Valid wake/sleep/non-wear/unknown minutes in the day		

### 2.1 Subjective sleep testing

Referring to previous literature (13–15), NHANES employs several validated questionnaire tools, such as sleep duration assessment and sleep disorders questionnaires to evaluate participants’ sleep patterns and quality.

Sleep Duration: The database includes self-reported sleep duration (in h for hours) in year cycles from 2005-2018. Participants are queried about their nightly sleep duration, providing a snapshot of how much sleep they typically receive. It should be noted that since 2017, the NHANES records sleep and wake time on weekdays and weekends, which allows more perspective to analyze the impact of weekend sleep supplementation on health outcomes.

Sleep Disturbances: NHANES delves into various sleep disturbances, encompassing issues like difficulty falling asleep, insomnia, sleep apnea, early morning awakenings, and instances of restless legs. This comprehensive evaluation allows for the identification of specific challenges individuals may face in achieving restful sleep and aids in identifying potential cases of obstructive sleep apnea. Moreover, NHANES assesses symptoms associated with Restless Leg Syndrome (RLS), including uncomfortable sensations in the legs, especially during periods of rest or inactivity. Understanding RLS prevalence provides valuable insights into this common sleep-related movement disorder.

Influence of Sleep Disturbances: This part includes different feelings of sleep disturbances, such as tiredness, leg jerks, and daytime sleepiness. Taking daytime sleepiness as an example, the questionnaire includes inquiries about daytime sleepiness, which can be indicative of sleep-related disorders or insufficient sleep duration. Understanding the influence of sleep troubles provides critical insights into the impact of sleep patterns on daily functioning.

### 2.2 Objective sleep testing

NHANES year cycles 2011-2014 introduced accelerometer-based sleep measurement such as physical activity monitors (PAM), which monitor movement and gravitational forces, providing data on physical activity levels, sedentary behavior, and sleep patterns.

There are multiple advantages to accelerometer-based sleep measurement. Unlike self-reported sleep data, accelerometry offers an objective and quantitative assessment of sleep parameters, reducing potential biases associated with subjective reporting. In addition, accelerometers enable continuous monitoring of sleep patterns (at least for one week), allowing for the assessment of changes over time. This objective measurement significantly enhances the accuracy and reliability of sleep-related information. There are several key sleep parameters obtained via accelerometry:

Sleep Timing: By assessing activity levels throughout the night, accelerometers provide information on sleep onset and offset times, aiding in the characterization of sleep-wake patterns.

Sleep Duration: Accelerometers provide a reliable estimate of total sleep duration. By analyzing periods of minimal movement, researchers can discern when individuals are in a state of restful sleep.

Sleep Efficiency: This metric assesses the percentage of time spent asleep while in bed. It helps differentiate between time spent in bed and actual time asleep, offering a more nuanced understanding of sleep quality.

Awakening Frequency: Accelerometry allows for the identification of awakenings during the night, providing valuable insights into sleep continuity and potential disruptions.

Sleep Onset Latency: This parameter measures the time it takes for an individual to transition from wakefulness to sleep once in bed. It offers crucial information on the ease of falling asleep.

In summary, the integration of accelerometers within the NHANES database represents a significant advancement in sleep research. This objective measurement approach enhances the precision and scope of sleep-related information, allowing for more robust analyses and deeper insights into the complex interplay between sleep and overall health in the American population. A recent publication has validated the single-item self-reported sleep duration in NHANES by comparing it with wrist-worn accelerometer data. The study suggested that associations between sleep duration and other health outcomes identified using NHANES data should undergo further examination using more accurate and valid measures of sleep duration (16, 17). However, one limitation worth noting is that relying solely on accelerometers does not allow for the differentiation between different sleep stages, such as rapid eye movement (REM) sleep and non-rapid eye movement (NREM) sleep. To gain a more comprehensive understanding of sleep patterns and their impact, polysomnography (PSG) emerges as a valuable tool, particularly in populations with shortened sleep durations, enabling a deeper insight into sleep architecture and stage distribution.

## 3 Future perspectives and conclusions

From the perspective of the association between sleep and chronic diseases, leveraging NHANES, researchers can delve into the relationship between sleep and chronic diseases such as cardiovascular ailments and diabetes. Studies have revealed a positive correlation between inadequate sleep and the incidence of chronic diseases (5–9), underscoring the significance of maintaining good sleep for prevention and management. Additionally, NHANES offers a wealth of mental health information, including data on depressive symptoms and anxiety levels. Research indicates a close link between sleep quality and mental well-being, with poor sleep potentially acting as a significant factor in various mental health issues (18). Currently, there is also evidence that environmental exposure, nutritional status, and physical activity all have influence on sleep health (19–22). Furthermore, NHANES permits researchers to conduct sleep studies on specific demographic groups such as children, adolescents, and the elderly (23–25). By comparing sleep characteristics across different age groups, a more comprehensive understanding of the impact of sleep at various life stages can be gained. However, NHANES was a cross-sectional design, which limited causal interference. Thus, further longitudinal studies are warranted to assess the association between sleep patterns and various health outcomes over time.

In conclusion, NHANES stands as a robust resource for investigating the relationship between sleep patterns and overall health. Its extensive data repository offers a myriad of opportunities for researchers to delve into this critical intersection. With the continued refinement of methodologies and the incorporation of advanced assessment tools, NHANES is poised to play an even more significant role in advancing our understanding of sleep and health.

## Author contributions

YY: Writing – review & editing, Writing – original draft, Validation, Supervision, Methodology, Investigation, Funding acquisition, Formal analysis, Conceptualization. YC: Writing – review & editing, Writing – original draft, Validation, Methodology, Investigation, Formal analysis, Conceptualization. MW: Conceptualization, Investigation, Formal analysis, Methodology, Validation, Writing – review & editing.
